# Supplementary figures and images for: Genome-Wide Analysis of microRNAs and Their Target Genes in Dongxiang Wild Rice (Oryza rufipogon Griff.) Responding to Salt Stress
Source: Int J Mol Sci. 2023 Feb 17;24(4):4069. doi: 10.3390/ijms24044069 (PMC9960954; doi:10.3390/ijms24044069)

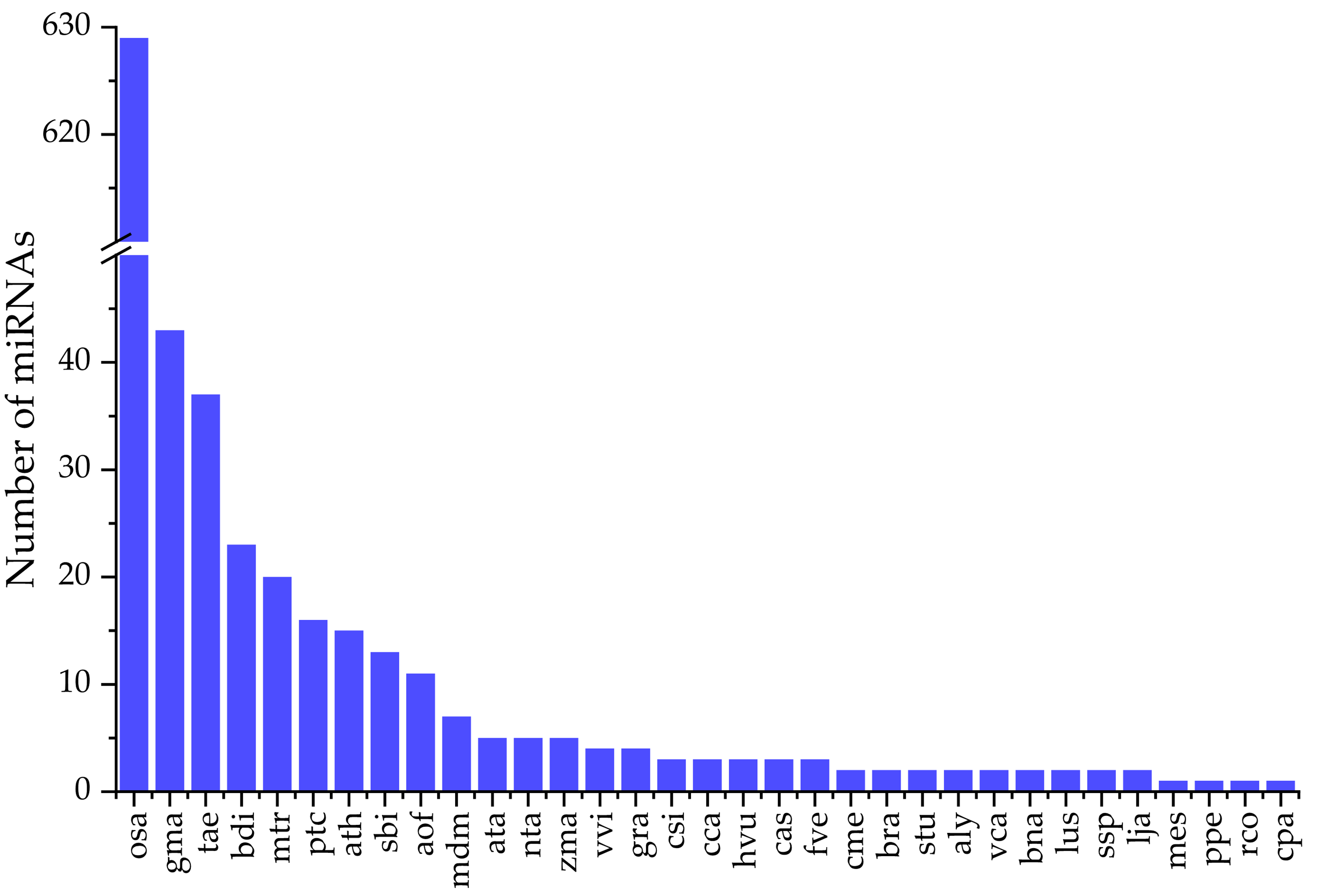

Supplement: Supplementary file 1 [file ijms-24-04069-s001.zip › Figure S1.tif]

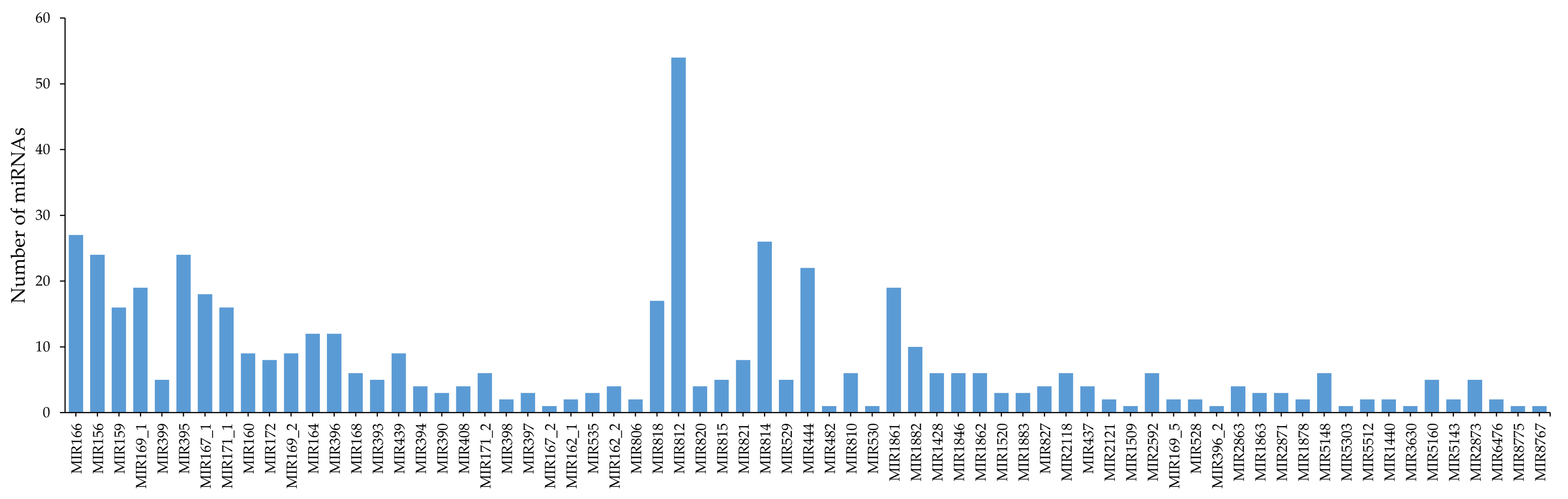

Supplement: Supplementary file 1 [file ijms-24-04069-s001.zip › Figure S2.tif]

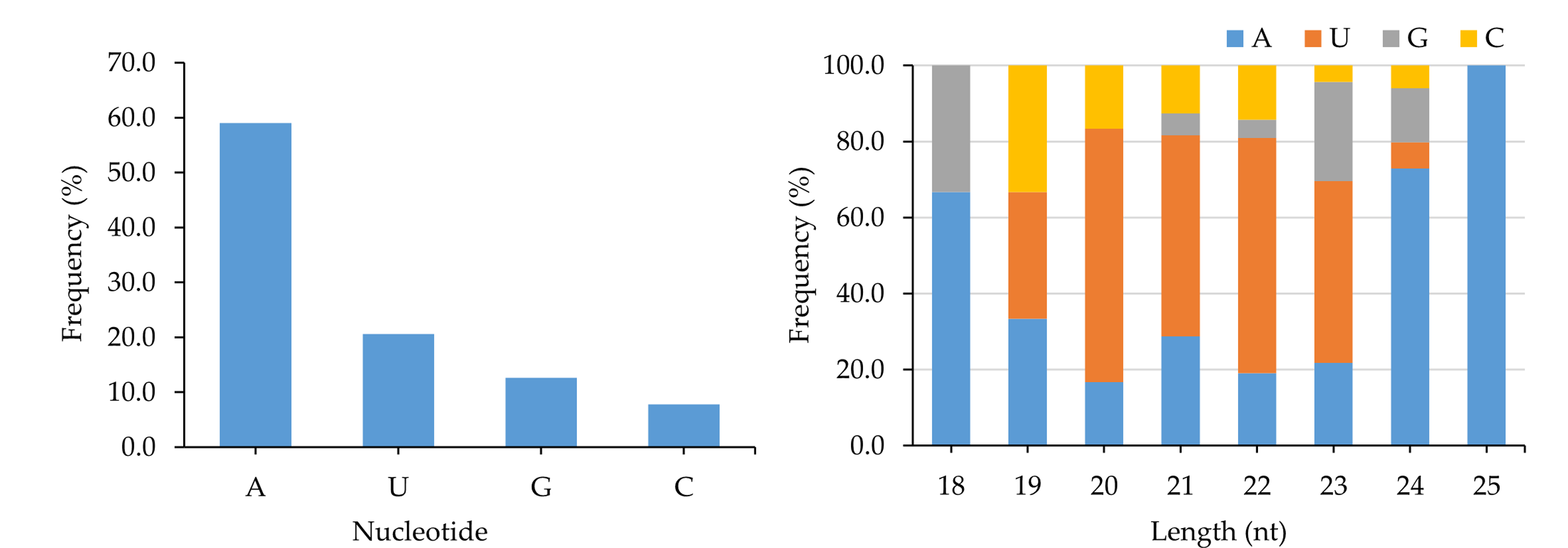

Supplement: Supplementary file 1 [file ijms-24-04069-s001.zip › Figure S3.tif]
